# Narrative competence disparities between Children’s hospital and General hospital in China: A comparative survey

**DOI:** 10.1371/journal.pone.0310375

**Published:** 2024-11-07

**Authors:** Junjun Jia, Rong Zhang, Qi Jin, Qinghua Zhou, Yun Xu

**Affiliations:** 1 Division of Hepatobiliary and Pancreatic Surgery, Department of Surgery, The First Affiliated Hospital, Zhejiang University School of Medicine, Hangzhou, China; 2 Chengde Medical University, Chengde, China; 3 Department of Nursing, International Healthcare Center, The First Affiliated Hospital, Zhejiang University School of Medicine, Hangzhou, China; Khyber Medical University, PAKISTAN

## Abstract

**Background:**

Narrative medicine was introduced in China in 2011 and has been applied as a tool for humane medical practice. The prominent problem in the narrative medicine is the lack of adequate attention and devotion. This study aimed to investigate Chinese medical staffs’ narrative competence and the influencing factors, confirming whether the level of narrative competence is different in different hospital settings.

**Methods:**

A cross-sectional survey was conducted among 1003 medical staffs, including 201 from Children’s hospital and 802 from the General hospital. The participants were scored based on the Chinese narrative competence scale, a brief Chinese version of the resilience scale, and a Chinese version of the self-efficacy scale. Data were analyzed using IBM SPSS version 25.0.

**Results:**

A total of 1003 medical staff from Children’s hospital and General hospital participated in the survey, with a response rate of 94.36%. Our results showed that the score of narrative competence of General hospital and Children’s hospital was 149.45±26.22 and 147.10±18.87, respectively, both of which were in intermediate level. Resilience, familiarity with narrative medicine were influencing factors of narrative competence in 2 kinds of hospitals, and whether having written parallel charts before were the influencing factors of narrative competence in General hospital. Besides, our study found that the level of narrative competence (χ^2^ = 13.672, p≤0.001), resilience score (personal ability dimension, t = 3.439, p≤0.001) and self-efficacy (t = 1.976, p<0.005) are different between General and Children’s hospital.

**Conclusions:**

Narrative competence is different between General hospital and Children’s hospital. Medical staff in General hospital are more familiar with narrative medicine. The competence of medical staff in China needs to be improved.

## 1. Introduction

Rita Charon first proposed the concept of narrative medicine in 2001, also known as medicine practiced with narrative competence [1]. The basis of the model is empathy, reflection, professionalism, and trust applied to clinical practice. Narrative competence is the ability to acknowledge, absorb, interpret, and act on the stories and plights of others, allowing medical staff to understand patients’ feelings and deliver appropriate and targeted help [2]. Medical staff and patients are an alliance to fight against the disease, because they are “schicksalsgemeinschaft”, a German word that means “a community of destiny”. This way, medical staff can understand the patients’ narration and suffering. Professor Liping Guo translated Charon’s book into Chinese and introduced narrative medicine to China, which attracted a growing number of researchers in narrative medicine [3–7].

Narrative medicine can strengthen doctor-patient relationships [1, 8], promote medical staffs’ empathic ability and professional achievement [9–11], mitigate the doctor-patient relationships [1, 12], and enable medical staffs to recognize their journeys through medicine [13–15]. Narrative competence is the intrinsic motivation and an essential ability for medical staff to achieve narrative medicine. Research has shown that the medical staffs’ empathy and humanistic care abilities had been improved through narrative medicine training, which enables medical staff to consider patients’ situations from multiple perspectives [5, 6, 16]. Medical students can analyze people and situations from various angles to gain a deeper, more profound understanding of human experiences using reflective thinking that are essential content of narrative medicine training [16]. Additionally, previous research found that age [17, 18], working length [3, 18, 19], familiarity with narrative medicine or nursing [3, 18, 19] are the key influencing factors of narrative competence. Those working longer with adequate narrative medicine or nursing knowledge would perform better in narration.

Resilience is considered a personality trait or a dynamic process refers to positive adaptation or the ability to maintain mental health when experiencing adversity in any situation [20]. Medical staff possess high level of resilience is crucial to perform professionally manage work, and can improve individuals solving problems, coping with stress, and staying motivated for career development [21]. Self-efficacy is not a skill but the confidence to reach the goal, defined as individuals’ beliefs about their ability to engage in actions required to get the desired goal [22]. Human behavior is result-oriented, nurses with high levels of self-efficacy achieve better narrative nursing [19]. In China, doctor-patient relationships seem to be exceptionally strained, and the reason might be the shortage of medical resources but, more profoundly, the growing demand for more personalized medical services [12]. Medical staff are the executors of narrative practice; the ability of medical staff to seek patients’ stories or narration and accept these as a resource in healthcare practices is critical to the recent development of person-centered care [23–25].

Medical staff face different types of patients in Children’s hospital and General hospital. In Children’s hospital, their patients are children, those who have less life experience, lower cognitive level, lower self-control ability, and lower cognitive degree of disease, resulting in significantly reduced compliance with disease treatment compared with adult patients. Besides, most children go to the hospital with at least one family member, in this point medical staff in Children’s hospital and General hospital meet the patients with different psychological characteristics, so we are wondering whether their narrative competence is the same.

In the present study, we conducted a cross-sectional survey of Chinese medical staff by online questionnaire to confirm the disparities of narrative competence of medical staff between Children’s hospital and General hospital.

## 2. Materials and methods

### 2.1 Participants

A convenience sample of clinical medical staff from General hospital and Children’s hospital in Zhejiang province, China, were enrolled in the study. Inclusion criteria were: (a) being working in hospital for more than 3 months, (b) deliver direct medical care to patients and written consent to participate.

Exclusion criteria were: (a) medical students, (b) medical staffs who did not deliver direct medical care to patients. The study was a cross-sectional online survey, conducted from 1^st^ December 2022 to 31^st^ March 2023. Data were collected using Questionnaire Star Software, widely used in China. IP-address-restriction technology was used to avoid ‘one person, multiple answers’. All participants were informed of the study purpose and provided informed consent (through an online form). The system had set all the options and were mandatory questions. Clinical medical staff received the survey link via WeChat (one of China’s most widely used social networking applications). The connection of this survey: https://www.wjx.cn/vj/woYS3wi.aspx.

### 2.2 Questionnaires

Our survey questionnaire includes four parts: sociodemographic information (e.g., age, gender, marital status, department, income, familiarity with narrative medicine or nursing), Narrative Competence Scale, Resilience Scale, and Self-efficacy Scale.

#### (1) Narrative Competence Scale (NCS)

The scale was a self-reporting scale to measure the medical staffs’ narrative competence and developed initially through literature, group discussion, and a questionnaire survey by Ma in 2019 [7], yielding a total of 27 items and divided into listening, understanding and reflection dimension. Each item in the scale was scored using a 7-point Likert scale, from 1 (strongly non-conformity) to 7 (strongly conformity), with a summed score ranging from 27 to 189 and classified into weak (<145), intermediate (145~163), strong (≥163) [7]. The Cronbach’s alphas for the entire and three dimensions were 0.950, 0.835, 0.912, and 0.842, respectively. The content validity for the full scale was 0.890, indicating appropriate stability and reliability of the scale [7].

#### (2) Resilience Scale (RS)

The RS-14 items short version of the Wagnild and Young RS-25 was used to measure the resilience of medical staff [26]. RS-14 is the widely used resilience scale divided into personal ability and positive perception dimensions. The scale was based on a 7-point Likert scale, from 1 (strongly nonconformity) to 7 (strongly conformity), and the total score ranged from 14 to 98. A higher total score or scoring rate indicated a higher level of resilience. However, there is no universally recognized cut-off to distinguish between low and high resilience. The internal consistency of the overall scale was 0.928, and the split-half reliability was 0.890, confirming the validity and reliability of the scale [26].

#### (3) Self-efficacy scale (SE)

We used the Chinese version of the Self-efficacy scale translated by Jia (Jia and Li, 2010), which was developed by Sherer [27]. It featured 17 items (6 items were positive, 11 were negative, and negative items scored in reverse). Each item was rated using a 6-point Likert scale, from 1 (strongly disagree) to 6 (strongly agree), and a total score ranging from 17 to 102. A higher total score or scoring rate indicated a higher level of self-efficacy. The split-half reliability was 0.71, and the content validity was 0.99, indicating appropriate stability and reliability of the scale [28].

### 2.3 Statistical analysis

Data were analyzed using IBM SPSS version 25.0. The basic demographic characteristics were demonstrated with mean ± (standard deviation) and frequency. The distribution of data was tested by F-test. The t-test and χ^2^ analysis were used to confirm the difference in the narrative competence, resilience, and self-efficacy level between two hospitals. P<0.05 is considered statistically significant.

### 2.4 Ethical considerations

The procedures followed in this study were approved by the Ethics Committee of the First Affiliated Hospital, Zhejiang University School of Medicine. All the data collected from the subjects were kept anonymous and confidential to protect their privacy.

## 3. Results

### 3.1 Level of medical staffs’ narrative competence with different characteristics and disparities of characteristics of Children’s hospital and General hospital

A total of 1063 questionnaires were filled out and collected. After preliminary screening, 60 questionnaires with illogical answers/missing data were screened out, leaving 1003 valid questionnaires with an effective rate of 94.36%, 802 medical staff from General hospital and the other 201 medical staff from Children’s hospital. Except for the gender and age, other characteristics of Children’s hospital and General hospital are different. All this information is listed in Table 1.

**Table 1:** Level of medical staffs’ narrative competence with different characteristics and disparities of characteristics of Children’s hospital and General hospital.

Variable		Level of NC in Children’s hospital					Level of NC in General hospital				χ^2^	p
	weak(n,%)	intermediate(n,%)	strong(n,%)	χ^2^	p	weak(n,%)	intermediate(n,%)	strong(n,%)	χ^2^	p		
Gender				2.057	0.358				2.489	0.288	0.041	0.840
Male	12(36.36)	14(42.42)	7(21.21)			54(42.52)	42(33.07)	31(24.41)				
Female	69(41.07)	79(47.02)	20(11.90)			239(35.41)	262(38.81)	174(25.78)				
Age				4.404	0.354				10.446	0.034	0.631	0.729
<30	15(41.67)	16(44.44)	5(13.89)			69(42.33)	57(34.97)	37(22.70)				
30~	56(44.09)	54(42.52)	17(13.39)			179(36.76)	174(35.73)	134(27.52)				
≥40	10(26.32)	23(60.53)	5(13.16)			45(29.61)	73(48.03)	34(22.37)				
Marital status				1.262	0.532				4.161	0.125	10.871	0.001
Single	13(46.43)	13(46.43)	2(7.14)			84(42.21)	72(36.18)	43(21.61)				
Married	68(39.31)	80(46.24)	25(14.45)			209(34.66)	232(38.47)	162(26.87)				
Education				2.427*	0.675				19.639	0.001	34.866	0.000
Junior college	2(66.67)	1(33.33)	0(0.00)			18(25.35)	33(46.48)	20(28.17)				
Bachelor	59(42.75)	61(44.20)	18(13.04)			233(37.89)	212(34.47)	170(27.64)				
Postgraduate	20(33.33)	31(51.67)	9(15.00)			42(36.21)	59(50.86)	15(12.93)				
Department				3.833	0.699				18.254	0.006	41.836	0.000
Internal Dept.	43(42.57)	48(47.52)	10(9.90)			129(36.24)	145(40.73)	82(23.03)				
Surgery Dept.	8(47.06)	7(41.18)	2(11.76)			78(33.48)	92(39.48)	63(27.04)				
ICU and ER	18(36.73)	21(42.86)	10(20.41)			56(50.45)	32(28.83)	23(20.72)				
Others	12(35.29)	17(50.00)	5(14.71)			30(29.41)	35(34.31)	37(36.27)				
Years of working (year)				2.722	0.843				21.095	0.002	8.943	0.030
<6	15(41.67)	15(41.67)	6(16.67)			72(46.45)	53(34.19)	30(19.35)				
6~	29(39.19)	36(48.65)	9(12.16)			72(34.12)	88(41.71)	51(24.17)				
11~	29(43.28)	28(41.79)	10(14.93)			117(36.34)	106(32.92)	99(30.75)				
21~	8(33.33)	14(58.33)	2(8.33)			32(28.07)	57(50.00)	25(21.93)				
Monthly income (RMB)				4.282	0.369				12.011	0.017	43.010	0.000
<5000	11(42.31)	11(42.31)	4(15.38)			108(36.49)	95(32.09)	93(31.42)				
5000~	51(45.13)	50(44.25)	12(10.62)			128(37.43)	135(39.47)	79(23.10)				
≥10000	19(30.65)	32(51.61)	11(17.74)			57(34.76)	74(45.12)	33(20.12)				
Profession				1.613	0.446				26.413	0.000	51.924	0.000
Doctor	44(37.29)	59(50.00)	15(12.71)			102(40.64)	114(45.42)	35(13.94)				
Nurse	37(44.58)	34(40.96)	12(14.46)			191(34.66)	190(34.48)	170(30.85)				
Professional qualification				5.048	0.282				11.119	0.025	20.376	0.000
Primary	24(43.64)	24(43.64)	7(12.73)			144(40.00)	119(33.06)	97(26.94)				
Intermediate	47(44.34)	45(42.45)	14(13.21)			110(34.59)	124(38.99)	84(26.42)				
Advanced	10(25.00)	24(60.00)	6(15.00)			39(31.45)	61(49.19)	24(19.35)				
Familiarity with narrative medicine				7.435	0.115				43.830	0.000	17.997	0.000
Never heard before	50(48.54)	39(37.86)	14(13.59)			133(43.46)	113(36.93)	60(19.61)				
Only heard before	27(32.93)	45(54.88)	10(12.20)			126(36.31)	144(41.50)	77(22.19)				
Skillful to practice	4(25.00)	9(56.25)	3(18.75)			34(22.82)	47(31.54)	68(45.64)				
Whether have read parallel chart before				2.682	0.262				22.695	0.000	13.241	0.000
Yes	11(32.35)	20(58.82)	3(8.82)			68(28.57)	83(34.87)	87(36.55)				
No	70(41.92)	73(43.71)	24(14.37)			225(39.89)	221(39.18)	118(20.92)				
Whether have written parallel chart before				3.835*	0.125				30.993	0.000	9.551	0.002
Yes	0(0.00)	4(80.00)	1(20.00)			9(12.50)	27(37.50)	36(50.00)				
No	81(41.33)	89(45.41)	26(13.27)			284(38.90)	277(37.95)	169(23.15)				

### 3.2 The ordinal logistic regression analysis

The ordinal logistic regression was conducted to establish the regression model of narrative competence. Resilience, self-efficacy, and those variables that would influence narrative competence were independent variables with narrative competence as a dependent variable. We found that resilience was an independent factor that influenced narrative competence in Children’s hospital, and independent factors that influenced narrative competence in General hospital were resilience, familiarity with narrative medicine, and whether have written parallel charts before (Tables 2 and 3).

**Table 2:** Ordinal regression of sociodemographic variables, resilience and self-efficacy in Children’s hospital (n = 201).

Variable	B	SE	Wald χ^2^	p	OR	95%CI	
						Lower	Upper
Resilience	0.151	0.019	63.099	0.000	1.163	1.121	1.208

**Table 3:** Ordinal regression of sociodemographic variables, resilience and self-efficacy in General hospital (n = 802).

Variable	B	SE	Wald χ^2^	p	OR	95%CI	
						Lower	Upper
Never heard narrative medicine before	-0.577	0.281	4.218	0.040	0.562	0.324	0.974
Never written parallel chart before	-0.876	0.305	8.263	0.004	0.417	0.229	0.757
Resilience	0.125	0.008	264.006	0.000	1.133	1.116	1.150

### 3.3 Disparities and scores of narrative competence, resilience and self-efficacy

The t-test or χ^2^ analysis showed that the level of narrative competence, resilience score (personal ability dimension) and self-efficacy are different between General and Children’s hospital (Table 4).

**Table 4:** Disparities of narrative competence of medical staff between Children’s hospital and General hospital.

Variable	(Mean±SD)/(n,%)		t/χ^2^	p
	Children’s hospital General hospital			
Narrative competence	147.10±18.87	149.45±26.22	13.672*	0.001
Weak	81(40.30)	293(36.53)		
Intermediate	93(46.27)	304(37.91)		
Strong	27(13.43)	205(25.56)		
Resilience	73.81±13.33	77.24±14.61	3.034	0.002
Personal ability	51.86±9.88	54.69±10.58	3.439	0.001
Positive perception	21.95±14.61	22.55±4.46	1.750	0.080
Self-efficacy	53.50±13.36	55.71±16.88	1.976	0.049

Note: *χ^2^-value

## 4. Discussion

We conducted a cross-sectional survey among 1003 medical staffs and found the narrative competence of medical staff in China is in intermediate level. Besides, independent factor that influenced narrative competence in Children’s hospital and General hospital was different. Meanwhile, the level of narrative competence, resilience score (personal ability dimension) and self-efficacy are different between General and Children’s hospital.

According to the NCS, narrative competence is classified into three levels: weak (<145), intermediate (145~163), and strong (≥163) [7]. Our study revealed that the general score of narrative competence of General hospital and Children’s hospital was 149.45±26.22 and 147.10±18.87 (intermediate level), respectively, similar to previous findings of medical staff in general hospitals in China [3, 18, 19, 29], indicating that the narrative competence of medical staff in China needs to be improved. Narrative medicine is a “new” conception for medical staff; in this study, almost 51.24% (from Children’s hospital) and 38.15% (from General hospital) participants had never heard of narrative medicine before, and only 16.92% (from Children’s hospital) and 29.68% (from General hospital) medical staffs had read parallel chart before, indicating that medical staffs knew little about narrative medicine. According to knowledge, attitude/belief, and practice (KAP) theory, knowledge is the foundation for practice, and lack of knowledge of narrative medicine might be the reason that eventually leads to a lower level of narrative competence of medical staff in this study. Previous reports [3, 17–19] suggest that reflective writing is one approach in facilitating practitioners’ reflection on the connection between their personal story and professional practices, while only 2.49% (from Children’s hospital) and 8.98% (from General hospital) medical staffs in our study had written parallel chart before. Reflective writing promotes self-reflection by putting thoughts and feelings into words [30]. They take note of the patients’ narration and deem this a helpful and achievable method. They would more likely anticipate integrated and complementary medical care, which means medical staff who are more familiar with narrative medicine do better in narrative practice. As we already know, narrative competence can be improved through training and practice [4, 5, 31–33]. In our study, 82.09% (Children’s hospital) and 80.67% (General hospital) participants had worked for more than 6 years. However, most of the medical staffs did not have the chance to know more about narrative medicine because most narrative curriculums are for medical students. Developing a comprehensive platform for a narrative competence training curriculum to promote the narrative practice and enrich the meanings of patients’ lives should be on the way.

Researchers found that resilience is positively correlated with narrative competence, which means that resilience might be used as an internal motivation to promote the narrative competence of medical staff [3, 34, 35]. Medical staff with good resilience are more enthusiastic and willing to devote the energy to meet the growing demand for patient-centered services and shared decision-making in a more diverse modern medical environment [31]. High self-efficacy can help nurses to achieve better narrative nursing [19]. Self-efficacy is an essential factor in narrowing the gap between knowledge and practice. Those with higher self-efficacy are more likely to find support from family members, colleagues and organizations, resulting in better performance in daily healthcare services. During the medical care duration, they become more confident in dealing with patients and meeting their needs, including medical services and emotional needs. Resilience and self-efficacy are different among medical staffs in Children’s hospital and General hospital, which might be another reason contributing to the difference in narrative competence.

According to the result of χ^2^ analysis, the narrative competence between Children’s hospital and General hospital is different, and *t*-test results showed that both resilience and self-efficacy are different between two hospitals. Data showed that the narrative competence of 81(40.30%) and 293(36.63%) medical staff are weak in Children’s hospital and General hospital, respectively. Table 3 shows that medical staffs in General hospital are more familiar with narrative medicine, more participants have read and wrote parallel chart before, which might be the reasons leading to the disparities between Children’s hospital and General hospital. Reading and writing a parallel chart is the foundation of narrative medicine, which more likely shows those medical staff have sufficient knowledge about narrative medicine so that the gap between knowing and doing can be narrowed and the narrative competence would be different. Consequently, those medical staff who had written parallel charts before would perform better narrative competence. Clinical medical staff who are more familiar with narrative medicine may do better in narrative practice.

## 5. Conclusion

Narrative medicine is the practice of medicine with narrative competence, which can provide a respectful environment that is empathic and wholesome. Our findings highlight that narrative competence of medical staff in China needs to be improved, and it is different between General hospital and Children’s hospital. Medical staff in General hospital are more familiar with narrative medicine. Parallel chart writing and resilience would impact the narrative competence of medical staff. To this end, developing a comprehensive platform for a narrative competence training curriculum to promote the narrative practice and enrich the meanings of patients’ lives should be on the way.

## 6. Limitation

There are several limitations to this study that should be considered when interpreting these results. Firstly, the study sample size was small and most are nurses, the conclusions and implications of the study are limited. Secondly, all the data used in this study were self-reported. Thirdly, narrative medicine is a tool for improving physician–patient relationship, beside narrative competence, many other factors influence the physician–patient relationship, such as social trust [36] and physician–patient communication [37], etc. In future studies, these factors should be included for analysis to draw a solid conclusion.

## Declarations

We used the Strengthening the Reporting of Observational Studies in Epidemiology (STROBE) guideline for cross-sectional studies. All methods were performed following the relevant guidelines and regulations.

## Supporting information

S1 Dataset General hospital and Children’s hospital. (ZIP)
